# Moderate-to-vigorous physical activity duration is more important than timing for physical function in older adults

**DOI:** 10.1038/s41598-020-78072-0

**Published:** 2020-12-07

**Authors:** Ting-Fu Lai, Yung Liao, Chien-Yu Lin, Wan-Chi Huang, Ming-Chun Hsueh, Ding-Cheng Chan

**Affiliations:** 1 Department of Health Promotion and Health Education, National Taiwan Normal University, Taipei, Taiwan; 2 Faculty of Sport Sciences, Waseda University, Tokorozawa, Japan; 3 Graduate School of Sport Sciences, Waseda University, Tokorozawa, Japan; 4 Graduate Institute of Sport Pedagogy, University of Taipei, Taipei, Taiwan; 5 Department of Geriatrics and Gerontology, National Taiwan University Hospital, Taipei, Taiwan; 6 Department of Internal Medicine, National Taiwan University Hospital, Taipei, Taiwan; 7 Superintendent Office, Chu-Tung Branch, National Taiwan University Hospital, Hsinchu County, Taiwan

**Keywords:** Public health, Health care

## Abstract

The positive association between the total duration of physical activity and performances of physical function may vary at different times of the day as circadian rhythm regulates individuals in response to external stimulations. We aimed to examine the association of timing-specific and overall moderate-to-vigorous physical activity (MVPA) with performances of physical function in older adults. A cross-sectional analysis was conducted among 118 older adults (mean age = 70.0 ± 5.0 years). We assessed and identified timing-specific (morning: 06:01–12:00; afternoon: 12:01–18:00; evening: 18:01–24:00) and overall MVPA using a triaxial accelerometer. Different measures of physical function were evaluated including handgrip strength (by grip dynamometer), gait speed (5-m walk test), basic functional mobility (timed up and go test), and lower limb strength (five times sit-to-stand test). Multivariate linear regression models adjusting for covariates were used to investigate the associations. Participants spent 25.0 (± 26.2) minutes in MVPA per day on average, half the time spent during the morning (47.7%), followed by during the afternoon (29.9%) and evening (21.6%). The time spent on overall MVPA was generally associated with better physical function performances. There was statistical evidence for the percentages of MVPA engagement during the morning [*B* = 0.214, 95% confidence interval (CI) 0.001 to 0.428] and afternoon (*B* = − 0.273, 95% CI − 0.518 to − 0.027) associated with basic functional mobility but with contrary directions; the percentage of MVPA engagement during the evening was associated with less time spent in gait speed performance (*B* = − 0.237, 95% CI − 0.468 to − 0.006). Our findings inform implications that the overall MVPA engagement was more important than timing-specific MVPA to older adults’ physical function performances. Strategies for accumulating time of MVPA is more practical and effective than encouraging to engage MVPA in specific timing for the enhancement of functional ability and therefore prevent disability among older adults.

## Introduction

Disability, a physical or mental condition that impairs or limits an individual’s movements in daily life, is one of the commonest symptoms in older populations. It was estimated that more than 46% of older adults aged 60 years or above have disabilities and more than 250 million older adults undergo moderate to severe disability in 2012^1^. It places not only a heavy burden of physical strain and emotional stress on the family caregiver^2^ but also an increase in medical care expenditure^3^. Several studies have shown that physical function is an important predictor of disability in older adults^4,5^. Previous research addressing older adults’ disability has shown that engaging sufficient levels of moderate-to-vigorous physical activity (MVPA) contributes to better performances of physical function (e.g., handgrip strength, gait speed, basic functional mobility, and lower limb strength)^6–8^.

There is a growing interest in establishing a link between the effects of circadian rhythms and sufficient physical activity in older adults^9^. An individual’s circadian rhythm is dynamic over his/her lifespan and deteriorates in the later years of life^9^; however, it still regulates individuals’ physiology and metabolism in response to external stimulations^10,11^. A systematic review showed that doing exercise, a specific form of regular physical activity, can lead to a phase shift of melatonin, thyroid-stimulating hormone and body temperature^12^. Furthermore, multiple external simulations such as sun exposure or fresh air may provide potential synergistic benefits and thus doing physical activity in the morning may be a strategy to maximally improve older adults’ physical function. Previous studies reported that older adults who were more active, indicated by the amount of physical activity or walking time, during the morning had lower risks of obesity^13^ and mortality^14^. Despite this, the relationship between timing-specific physical activity and physical function remains to be investigated. For accurately identifying the different timings of MVPA engagement during the day, the accelerometers are more favorable to be used than self-reported measures because they could track the orientation and movement in individuals objectively^15^.

We hypothesized that the MVPA engagement during the morning was associated with older adults’ physical function after controlling for its overall duration and therefore investigated the association of timing-specific and overall MVPA with a range of measures of physical function among community-dwelling older adults.

## Methods

### Participants

Older adults aged over 60 years who could walk independently were recruited using local advertisements and announcements from 28 different residential neighborhoods in Taipei City, Taiwan. Among these participants, 118 out of 130 older adults wearing the accelerometer for a consecutive seven days and completed the on-site examinations. A valid day was defined wearing accelerometer at least 10 h during waking hours and participants who had at least 4 valid days were included in all analyses. The inclusion criteria were widely used in previous studies^16,17^. There were no differences in sex and age in the included and excluded participants (data not shown). Detailed methods and procedures have been reported in a previous study using objectively measured data (n = 124)^17^.

### Outcome variables

We used on-site examinations to assess four measures of physical function, including handgrip strength, gait speed, basic functional mobility, and lower limb strength. The measurements have been widely used in previous studies^18^. We measured the handgrip strength of both of the participant’s hands by turns using the hydraulic hand dynamometer (Jamar Plus + Digital Hand Dynamometer, Lafayette Instrument Company, USA). A higher measure of handgrip strength indicated better performance, and the maximum strength measurement was selected from three attempts with a 1-min gap between attempts. Each participant had only one attempt to walk 11 m as fast as possible. The time spent walking in the central 5 m was calculated. A shorter time spent on the 5-m walk test indicated better performance of gait speed. In term of basic functional mobility, participants were instructed to rise from a standard chair, walk 3 m forward, return to the chair, and sit down as soon as possible. Each participant repeated the same attempt twice and the attempt with the shorter time, indicating better performance, was selected. Lower limb strength using the five times sit-to-stand test was also assessed. Participants were requested to sit on a standard chair and repeat stand up and sit down five times, as fast as they could. Each participant had two attempts and the attempt with the shorter time, indicating better performance, was selected.

### Exposure variables

Time spent in light physical activity (100–2019 counts/min) and MVPA (≥ 2020 counts/min) was measured by a waist-wear triaxial accelerometer (ActiGraph GT3X+, Pensacola, FL, USA)^20^. We followed the information on data collection and processing criteria suggested by a systematic review of standard protocols^19^. The different time intervals were identified in which the MVPA occurred: (a) morning: 06:01–12:00; (b) afternoon: 12:01–18:00; and (c) evening: 18:01–24:00 based on previous studies^14,20,21^. The accelerometer data showed around 90% of the participants in this study slept from 0:00 to 06:00 for each night and did not engage in MVPA. We calculated the percentage of the three time intervals of MVPA engagement for each participant. The ActiLife software version 6.0 was used to process the accelerometer data; all the data were processed by using 60-s epochs with default (i.e., 30 Hz) of sampling frequency.

### Covariates

The covariates including (1) sex, (2) age, (3) body mass index (BMI; calculated by weight in kilograms divided by height in meters squared), (4) marital status, (5) employment, (6) living status, (7) educational level, (8) self-rated health, (9) depression, (10) diabetes, (11) hypertension, (12) hyperlipidemia, (13) light physical activity, and (14) monitor wear time were assessed by an interviewer-administered questionnaire (1–12) and an accelerometer (13–14).

### Statistical analyses

We analyzed data from 118 older adults who completed all the study variables. We selected the covariates for different measures of the physical function using univariate linear regression models (Appendix Table 1). Unstandardized coefficients (*B*) were used to estimate the associations of the time spent in timing-specific and overall MVPA (in minutes) with measures of the physical function using multivariate linear regression models after adjusting for their covariates (i.e., the variables showing statistically significant in the univariate analyses). Log-transformed values of the study variables were used if the distributions of their raw values were skewed. R-squared was calculated to represent the proportion of the variance for an outcome variable that was explained by exposure variables in a regression model, namely the explanatory power of each model. All analyses were conducted in IBM SPSS Statistics 23.0 and the significance level was set at *p* < 0.05.

**Table 1:** Participants’ characteristics (n = 118).

Categorical variables	N	%
Sex: female	83	70.3
Marital status: married	78	66.1
Employment: no full-time job	114	96.6
Living status: living alone	11	9.3
Educational level: tertiary	26	22.0
Self-rated health: good	36	30.5
Depression: yes	16	13.6
Hypertension: yes	44	37.3
Hyperlipidemia: yes	35	29.7
Diabetes: yes	22	18.6

Continuous variables	Mean	SD
Age (year)	70.0	5.0
Body mass index (kg/m^2^)	24.2	3.5
Monitor wear time (hour/day)	15.4	1.4
Average daily steps^a^	7185.8	3527.4
Moderate physical activity (min/day)^a^	24.8	26.0
Vigorous physical activity (min/day)^a^	0.2	0.7
**Light physical activity**		
Overall (min/day)	290.9	83.6
During the morning^1b^ (%)	40.6	8.6
During the afternoon^2b^ (%)	35.1	6.3
During the evening^3b^ (%)	24.3	7.8
**Moderate-to-vigorous physical activity**		
Overall (min/day)	25.0	26.2
During the morning^1b^ (%)	47.7	16.1
During the afternoon^2b^ (%)	29.9	10.0
During the evening^3b^ (%)	21.6	11.1

SD: standard deviation.

^a^These variables were not considered as covariates.

^b^The sum of percentages of light physical activity and MVPA during the morning, afternoon, and evening may be not 100.0% because each percentage of time interval was an average of 118 participants.

^1^During the morning: 06:01–12:00.

^2^During the afternoon: 12:01–18:00.

^3^During the evening: 18:01–24:00.

### Ethics approval and consent to participate

This study was approved by the Research Ethics Committee of National Taiwan Normal University (REC Number: 201711HM003). Our study was conducted in accordance the 1975 Declaration of Helsinki and its subsequent revisions. The written informed consent was obtained from all subjects prior to participating in these studies.

## Results

The characteristics of 118 older adults (70.3% female; mean age = 70.0 years) were presented in Table 1. Participants spent 25 min in MVPA per day on average, almost half the time was during the morning (47.7%), followed by the time spent during the afternoon (29.9%) and evening (21.6%) (Table 1). A similar “morning occupying the most” pattern was also shown in light physical activity but the difference between different timings of the day was smaller than MVPA (Table 1).

Table 2 shows the associations of timing-specific and overall MVPA with four measures of physical function. In the adjusted models, overall MVPA duration was generally associated with better performances of handgrip strength (*B* = 0.045, 95% confidence interval [CI] 0.017 to 0.072), gait speed (*B* = − 0.061, 95% CI − 0.091 to − 0.031), and basic functional mobility (*B* = − 0.045, 95% CI − 0.079 to − 0.011). The associations of the percentages of MVPA during the morning (*B* = 0.214, 95% CI 0.001 to 0.428) and afternoon (*B* = − 0.273, 95% CI − 0.518 to − 0.027) were associated with basic functional mobility, respectively. However, their associations were in contrary directions. Additionally, there was statistical evidence for the percentage of MVPA during the evening was associated with better performance of gait speed (*B* = − 0.237, 95% CI − 0.468 to − 0.006). The R-squared statistics of the model using overall MVPA duration were generally larger than the models using percentage of MVPA engagement across different timings of the day in the four measures of physical function.

**Table 2:** Associations of overall and timing-specific moderate-to-vigorous physical activity with measures of physical function.

Measures of physical function	Overall MVPA			Percentage of overall MVPA								
				During the morning (06:01–12:00)			During the afternoon (12:01–18:00)			During the evening (18:01–24:00)		
	*B*	95% CI	*p*	*B*	95% CI	*p*	*B*	95% CI	*p*	*B*	95% CI	*p*
Handgrip strength (kg)^a^	0.045	(0.017, 0.072)	0.002*	− 0.069	(− 0.243, 0.106)	0.436	0.067	(− 0.132, 0.266)	0.504	− 0.003	(− 0.208, 0.201)	0.976
R-squared		0.640			0.598			0.598			0.596	
Gait speed (s)^b^	− 0.061	(− 0.091, − 0.031)	< 0.001**	0.075	(− 0.021, 0.270)	0.452	− 0.038	(− 0.263, 0.187)	0.738	− 0.237	(− 0.468, − 0.006)	0.044*
R-squared		0.433			0.349			0.346			0.370	
Basic functional mobility (s)^b^	− 0.045	(− 0.079, − 0.011)	0.009*	0.214	(0.001, 0.428)	0.049*	− 0.273	(− 0.518, − 0.027)	0.030*	− 0.162	(− 0.416, 0.092)	0.210
R-squared		0.368			0.351			0.356			0.337	
Lower limb strength (s)^b^	− 0.037	(− 0.081, 0.006)	0.094	− 0.048	(− 0.323, 0.226)	0.729	− 0.087	(− 0.399, 0.226)	0.583	0.205	(− 0.112, 0.521)	0.202
R-squared		0.164			0.144			0.145			0.155	

*B* unstandardized linear regression coefficient, *MVPA* moderate-to-vigorous physical activity, *CI* confidence interval.

^a^A positive association indicates better physical function accompanied by more time spent in MVPA.

^b^A positive association indicates worse physical function accompanied by more time spent in MVPA.

*p < 0.05; **p < 0.001.

## Discussion

Our data showed that the engagement in longer MVPA was generally associated with better performances of physical function and some statistical evidence for associations between specific-timing MVPA and physical function in older adults. In keeping with previous studies suggesting the engagement of MVPA could retain muscle density and function in older adults^6,7,22^, positive associations of the overall duration of MVPA with performances of physical function were observed. By contrast, our data did not show the benefits of physical function from higher levels of MVPA during the morning, inconsistent with previous findings^13,14^. Interestingly, there were inconsistent associations of the MVPA engaging in the morning and afternoon with the performance of basic functional mobility. Some studies suggested that the increased levels of serum vitamin D, which could be synthesized by sun exposure^23^, could directly strengthen older adults’ bones and muscles^24^ as well as indirectly improve their muscle strength through promoting sleep quality of older population^25,26^. However, our result regarding the MVPA engagement during the morning and the performance of basic functional mobility did not fully support the synergistic benefits from sun exposure. There may be other factors that may influence this association. For example, a pilot showed that exercising in the fasted or fed state in the morning would have different effects on facilitating adaptations in muscle^27^.

There was a significant association between the MVPA engagement during the evening and the performance of gait speed. The positive association between evening MVPA engagement and gait speed may be due to stronger intent of home-based walking at night within a safer neighborhood. Previous studies showed that older adults who perceived themselves living in safer neighborhoods (e.g., lower levels of crime and more comprehensive crossings) were more likely to be associated with more time in MVPA^28^ and daily step counts^17^ and lead to better physical function^29^. Future studies assessing a range of potentially influencing factors such as the state of exercise and perceptions of neighborhood safety should investigate the underlying mechanism of these associations.

Despite some associations showed between the timing-specific MVPA and physical function, it seemed that the duration of MVPA engagement across the time of the day was more important to older adults’ performances of physical function. Our findings imply that older adults accumulate more duration of MVPA, even through interrupted sections, may have better performances of physical function, irrespective of the timing of the day for MVPA engagement.

To our knowledge, this is the first study examining the association between the time spent in MVPA at different times of the day and physical function in the context of older adults. Furthermore, we used an accelerometer to objectively measure MVPA and provide such robust data. There were some limitations to this study. First, the association of the percentages or duration of timing-specific and overall MVPA with physical function should be interpreted with caution, as the size of our study was small. Future studies using a larger sample size are warranted to examine the associations between timing-specific versus overall MVPA with physical function in older adults. Second, although we regarded the same period of the day for each participant as sleep duration (i.e., 00:00–06:00) so those older adults would not engage in physical activity at that period, there may be some inconsistency of lifestyle between participants and this may cause bias. However, our data showed that the sleep periods in around 90% of the participants highly overlapped. Third, we did not use a standardized procedure to screen the cognitive ability of participants before the on-site examinations; therefore, there may be a marked variation in an understanding and reaction to the examinations in each older adult. The variation in the levels of cognition may affect their performances of physical function and confound the associations observed. Finally, the associations of the percentages and duration of timing-specific and overall MVPA with physical function cannot be extrapolated as causal relationships because of the cross-sectional design.

## Conclusion

This study suggests that engaging in more MVPA, regardless of the specific time of the day, is associated with better physical function performances among older adults. Designing strategies/programs with increasing the duration of MVPA engagement across the time of the day could benefit older adults’ physically functional ability and may, therefore, prevent disability.

## Supplementary information

Supplementary Information.

## Data Availability

Data used in this study are available upon reasonable requests.
