# Supplementary material for: Moderate-to-vigorous physical activity duration is more important than timing for physical function in older adults
Source: Sci Rep. 2020 Dec 7;10:21344. doi: 10.1038/s41598-020-78072-0 (PMC7721720; doi:10.1038/s41598-020-78072-0)
Supplement: Supplementary file 1 — Supplementary Information. [file 41598_2020_78072_MOESM1_ESM.docx]

Moderate-to-vigorous physical activity duration is more important than timing for physical function in older adults

Ting-Fu Lai ^1^, Yung Liao ^1, 2^, Chien-Yu Lin ^3^, Wan-Chi Huang ^1^, Ming-Chun Hsueh ^4^, Ding-Cheng Chan ^5,6,7§^.

^1^  Department of Health Promotion and Health Education, National Taiwan Normal University, Taipei, Taiwan

^2^ Faculty of Sport Sciences, Waseda University, Tokorozawa, Japan

^3^ Graduate School of Sport Sciences, Waseda University, Tokorozawa, Japan

^4^ Graduate Institute of Sport Pedagogy, University of Taipei, Taipei, Taiwan

^5^  Department of Geriatrics and Gerontology, National Taiwan University Hospital, Taipei, Taiwan

^6^ Department of Internal Medicine, National Taiwan University Hospital, Taipei, Taiwan

^7^ Superintendent Office, Chu-Tung Branch, National Taiwan University Hospital, Hsinchu County, Taiwan

^§^Corresponding author:

Ding-Cheng Chan

Superintendent Office, National Taiwan University Hospital Chu-Tung branch

E-mail: dingchengchan@ntu.edu.tw

Tel.: +886-3-5967668

Target Journal: Scientific Reports

Article Type: Short paper

# Appendix Table 1. The univariate linear regression models with measures of physical function

| **Categorical variables** | Handgrip strength ^a^ | Gait speed ^b^ | Basic functional mobility ^b^ | Lower limb strength ^b^ |  |
| --- | --- | --- | --- | --- | --- |
|  | *B* (95% CI) | *B* (95% CI) | *B* (95% CI) | *B*  (95% CI) | |
| Sex: female | -0.186 (-0.218, -0.155)* | 0.043 (0.004, 0.082)* | 0.013 (-0.031, 0.057) | -0.014 (-0.064, 0.037) | |
| Marital status: married | -0.059 (-0.103, -0.016)* | 0.050 (0.013, 0.088)* | 0.039 (-0.003, 0.080) | 0.013 (-0.036, 0.062) | |
| Employment: no full-time job | 0.101 (-0.015, 0.217) | -0.020 (-0.120, 0.080) | -0.058 (-0.168, 0.053) | -0.049 (-0.177, 0.079) | |
| Living status: living alone | 0.041 (-0.032, 0.113) | -0.022 (-0.084, 0.041) | -0.031 (-0.100, 0.038) | -0.082 (-0.161, -0.004)* | |
| Educational level: tertiary | 0.025 (-0.031, 0.081) | 0.071 (0.029, 0.113)* | 0.066 (-0.019, 0.122)* | 0.025 (-0.031, 0.081) | |
| Self-rated health: good | -0.053 (-0.099, -0.008)* | 0.056 (0.019, 0.094)* | 0.071 (0.030, 0.113)* | 0.081 (0.033, 0.130)* | |
| Depression: yes | 0.077 (0.017, 0.138)* | -0.064 (-0.116, -0.013)* | -0.076 (-0.133, -0.019)* | -0.061 (-0.128, 0.006) | |
| Hypertension: yes | -0.005 (-0.049, 0.039) | -0.038 (-0.075, -0.001)* | -0.040 (-0.081, 0.001) | -0.031 (-0.079, 0.017) | |
| Hyperlipidemia: yes | 0.014 (-0.033, 0.060) | -0.045 (-0.084, -0.006)* | -0.053 (-0.096, -0.010)* | -0.037 (-0.087, 0.014) | |
| Diabetes: yes | 0.024 (-0.031, 0.078) | -0.030 (-0.076, 0.017) | -0.051 (-0.102, -0.001)* | -0.019 (-0.079, 0.040) | |
| **Continuous variables** | *B* (95% CI) | *B* (95% CI) | *B* (95% CI) | *B* (95% CI) | |
| Age (year) | -1.050 (-1.726, -0.373)* | 1.220 (0.665, 1.775)* | 1.271 (0.649, 1.893)* | 0.960 (0.212, 0.463)* | |
| Body mass index (kg/m^2^) | 0.130 (-0.216, 0.476) | 0.502 (0.222, 0.782)* | 0.480 (0.165, 0.796)* | 0.219 (-0.158, 0.596) | |
| Monitor wear time (hours/day) | -0.019 (-0.035, -0.004)* | -0.006 (-0.019, 0.007) | -0.001 (-0.016, 0.014) | -0.006 (-0.023. 0.011) | |
| Light physical activity (hours/day) | -0.012 (-0.028, 0.003) | -0.009 (-0.022, 0.005) | -0.010 (-0.025, 0.004) | -0.014 (-0.031, 0.002) | |

*B*: unstandardized linear regression coefficient; CI: confidence interval

**p* < 0.05

^a^ a positive association indicates better physical function accompanied by meeting the characteristic or more quantity they had.

^b^ a positive association indicates worse physical function accompanied by meeting the characteristic or more quantity they had.
